# Generation and Characterization of the First Immortalized Alpaca Cell Line Suitable for Diagnostic and Immunization Studies

**DOI:** 10.1371/journal.pone.0105643

**Published:** 2014-08-20

**Authors:** Valentina Franceschi, Sarah Jacca, Elena L. Sassu, Fabio F. Stellari, Vicky L. van Santen, Gaetano Donofrio

**Affiliations:** 1 Department of Medical Veterinary Science, University of Parma, Parma, Italy; 2 Department of Pathobiology, College of Veterinary Medicine, Auburn University, Auburn, Alabama, United States of America; University of Liverpool, United Kingdom

## Abstract

Raising of alpacas as exotic livestock for wool and meat production and as companion animals is growing in importance in the United States, Europe and Australia. Furthermore the alpaca, as well as the rest of the camelids, possesses the peculiarity of producing single-chain antibodies from which nanobodies can be generated. Nanobodies, due to their structural simplicity and reduced size, are very versatile in terms of manipulation and bio-therapeutic exploitation. In fact the biotech companies involved in nanobody production and application continue to grow in number and size. Hence, the development of reagents and tools to assist in the further growth of this new scientific and entrepreneurial reality is becoming a necessity. These are needed mainly to address alpaca disease diagnosis and prophylaxis, and to develop alpaca immunization strategies for nanobody generation. For instance an immortalized alpaca cell line would be extremely valuable. In the present work the first stabilized alpaca cell line from alpaca skin stromal cells (ASSCs) was generated and characterized. This cell line was shown to be suitable for replication of viruses bovine herpesvirus-1, bovine viral diarrhea virus and caprine herpesvirus-1 and the endocellular parasite *Neospora caninum*. Moreover ASSCs were easy to transfect and transduce by several methods. These two latter characteristics are extremely useful when recombinant antigens need to be produced in a host homologous system. This work could be considered as a starting point for the expansion of the biotechnologies linked to alpaca farming and industry.

## Introduction

Two domestic forms of South American camelids are recognized today, the alpaca (*Lama pacos L.*) and the llama (*Lama glama L.*) whose evolutionary origins are debated [1]. Alpacas are the most abundant and the popularity of alpacas as agricultural and companion animals is increasing, with a population of over 3 million animals worldwide, used for fiber, meat production and packing [2]. The alpaca industry in the United States began when these camelids were first imported in 1984 from South America. Since this time owners, breeders, and speculators alike have been promoting the industry, pushing for national herd expansion to facilitate a base of animals adequate to sustain a viable alpaca textile industry. Moreover alpaca breeding programs are growing in other countries like Canada, Australia, and European countries.

When exotic species are introduced, they are potentially susceptible to indigenous pathogens causing more severe disease than in the natural host. Alpaca outside their native South American habitat are exposed to pathogens endemic to the domestic farm animals to which alpaca herds often have close proximity. Conversely, newly introduced alpaca could potentially deliver microorganisms not pathogenic to them but highly pathogenic to the indigenous animals. This situation can be very risky when newly introduced and indigenous animals are raised in close proximity allowing their pathogens to be exchanged with each other. Furthermore, because many alpaca are kept as companion animals the possibility of pathogen transmission between alpacas and humans should not be ignored. An example would be tuberculosis, reported many times in alpaca and llama [3]–[5]. Thus new reagents for diagnostic use, to prevent and combat potential alpaca pathogens are needed.

Despite these health issues, which could be considered a drawback for alpaca farming, alpaca, like other camelids, have the unique property of producing homodimeric single chain antibodies in their blood [6], [7]. Recombinant nanobodies derived from these single chain antibodies are small, bind targets with nM affinity, are stable and easy to manipulate. Moreover, the nanobodies often bind to epitopes that are less immunogenic for conventional antibodies, such as the active sites of enzymes. Due to their small size, they also target areas that are not accessible to standard antibodies [6], [7]. In the present paper we report the establishment and characterization of the first immortalized alpaca cell line. This cell line can be extremely useful to produce and carry antigen cargo for alpaca immunization and nanobody production, as a diagnostic tool for alpaca pathogen isolation and as a source of cell components for comparative and basic studies.

## Materials and Methods

### Organotypic skin cultures from alpaca

Skin biopsy specimens were collected from two clinically healthy 1-year-old female alpacas (Vicugna pacos) in the ‘Alpaca el coto’ herd, a private land located in Varano de’ Melegari [Parma, Italy (google map: https://www.google.com/maps/@44.704188,10.000825,14z?hl=it-IT)]. This sample collection did not involve endangered or protected species and no specific permissions were required for these locations/activities.

The samples were taken with a 6 mm biopsy punch from lateral neck, after hair removal and disinfection of the surgical site using 4×4 gauzes soaked with povidone-iodine solution. Animals were then anesthetised with 2% lidocaine, locally administered with a 20 *ga* needle. Once the punch biopsy instrument had penetrated the dermis and reached the hub, the biopsy was removed. An absorbable haemostatic gauze was applied to the wound in order to create good haemostasis and promote healing. Each explant was immediately transferred into complete medium [Eagle’s Modified Eagle Medium (EMEM) containing 20 µg/mL chloramphenicol, 50 IU/mL penicillin, 50 µg/mL streptomycin and 2.5 µg/mL amphotericin B) and 20% fetal bovine serum (FBS)] and kept on ice. Then the skin sections were washed several times in complete medium and cut into small slices. These slices were transferred to six-well tissue culture plates, so that each well contained a single piece of tissue, and then were wetted by a quantity of complete medium enough to slightly cover them. Culture plates were incubated for 10 days at 37°C in a humidified atmosphere with 5% CO_2_ in air. The culture medium was changed every 24 h. When cells growing around the slice were microscopically appreciable, slices were removed and the cells allowed to grow until semi-confluent. Then cultures were trypsinised, resulting in isolated cells that were transferred to flasks; the culture medium was changed every 48 h until the primary cells reached confluence. All cultures were maintained at 37°C with 5% CO_2_ in air in a humidified incubator.

### Alpaca cell transfection, selection and immortalization

Second-passage alpaca primary cells from a sub-confluent 75 cm^2^ flask were electroporated (Equibio apparatus; 300 V, 25 µF, 240 V, 1050 µF, and 481 R; Opty-Pulse) with 10 µg of pSV40T/neo [8] (a gift from Dr. Katerina Gordon, Beatson Institute, Glasgow, U.K.) DNA in Dulbecco’s Modified Eagle Medium with high glucose (DMEM high) with 10% FBS. Electroporated cells were transferred to new 75 cm^2^ flasks and fed with complete medium (EMEM containing 10% FBS, 50 IU/mL of penicillin, 50 µg/mL streptomycin, 2.5 µg/mL amphotericin B, and 2 mM L-glutamine). Twenty-four h after electroporation, stably transfected cells were selected with 700 µg/mL of G418 (Sigma) until visible colonies appeared on the surface of the flask. Three selected clones were independently passaged 100 times in the presence of G418. Thus, SV40 large T antigen immortalized alpaca skin stromal cell lines (ASSCs) were obtained.

### Cell lines

Bovine embryo kidney [(BS CL-94) BEK, from M. Ferrari, Istituto Zooprofilattico Sperimentale, Brescia, Italy], Madin Darby Bovine Kidney (MDBK, ATCC: CCL-22), African green monkey kidney epithelial cells [(VERO, ATCC: CCL-81) generously provided by Professor S. Trees, University of Liverpool] and Alpaca Skin Stromal cells (ASSC) were cultured in EMEM (Lonza) containing 10% FBS, 2 mM L-glutamine (SIGMA), 100 IU/mL penicillin (SIGMA) and 100 µg/mL streptomycin (SIGMA).

### Western immunoblotting

Cell extracts were obtained from ASSCs scraped or trypsinized from 25 cm^2^ confluent flasks at several different passage levels (from 5^th^ to 60^th^) by adding 100 µL of cell extraction buffer (50 mM Tris–HCl, 150 mM NaCl, and 1% NP-40; pH 8) to cell pellets. Cell extracts containing 50 µg of total protein were electrophoresed through sodium dodecyl sulfate-8% polyacrylamide gels and transferred to nylon membranes by electroblotting. Membranes were incubated with mouse anti-SV40 large/small T antigen monoclonal antibody (sc-58665; Santa Cruz Biotechnology Inc.), which was detected with horseradish peroxidase-labelled goat anti rabbit immunoglobulin G1 (IgG1) antibody (A0545; Sigma), and visualized by enhanced chemiluminescence (ECL Kit; Pierce).

### Alpaca cell growth assay

ASSCs at the 60^th^ passage were seeded into 6 well plates (5×10^4^ cells/well) and incubated at 37°C with 5% CO_2_ in air in a humidified incubator. Every 24 h cells were trypsinised and counted in triplicate. The results were analysed by an exponential regression method (http://mathworld.wolfram.com/LeastSquaresFittingExponential.html) to estimate ASSC’s doubling time.

### Cell immunostaining

ASSCs at the 3^rd^, 10^th^, 20^th^, or 60^th^ passage were seeded into a 6 well plate (2.5×10^5^ cells/well) and incubated at 37°C with 5% CO_2_ in air in a humidified incubator. When ASSCs were sub-confluent the culture medium was removed and the cells were fixed with acetone/methanol solution (1∶1) for 20 min at room temperature (RT). After two quick washes with phosphate buffered saline (PBS) the fixed cells were blocked for 1 h at room temperature with 10% FBS diluted in PBS +1% Bovine serum albumin (BSA). Subsequently cells were washed with PBS and incubated with the primary antibodies: anti α-vimentin mouse monoclonal antibody (sc-32322, Santa Cruz Biotechnology Inc.) diluted 1∶200, anti cytokeratin 14 (CK14) rabbit polyclonal antibody (PRB-155P, Covance) diluted 1∶500 or anti bovine CK18 mouse monoclonal antibody (KS-B17.2, Sigma) diluted 1∶200 in PBS+BSA1% for 1 h at RT. The antibody was then removed and the cells washed extensively with PBS three times for 3 min each. Cells were incubated with the secondary antibodies: Alexa 488-conjugated goat anti-mouse IgG (A11029, Life Technologies), AlexaFluor 594-conjugated goat anti-mouse IgG (A11032, Life Technologies) or AlexaFluor 594-conjugated goat anti-rabbit IgG (A11037, Life Technologies), each diluted 1∶500 in PBS+1%BSA for 1 h at RT in the dark and after that washed three times with PBS. These antibodies were previously validated for bovine specimens [9]. As a counterstaining 4′,6-diamidino-2-phenylindole (DAPI) was added to the cells and incubated for 10 min in the dark; after a final PBS washing cells were observed at the microscope.

### Transfection assay

ASSCs were seeded into 6 well plates (2.5×10^5^ cells/well) and incubated at 37°C with 5% CO_2_ in air in a humidified incubator. When ASSCs were sub-confluent the culture medium was removed and the cells were transfected with pEGFP-C1 (Clontech) using various transfection reagents.

Three µg of pEGFP-C1 were mixed with 9 µL of Fugene HD transfection reagent (Promega) in 150 µL of DMEM high without serum. After 15 min of incubation at RT, 850 µL of medium with 10% FBS were added and the transfection solution was added to cells. For LTX-mediated transfection 3 µg of pEGFP-C1 were mixed with 7 µL of LTX (Invitrogen), according to the manufacturer’s instructions, in 2 mL of DMEM high without serum; after 15 min of RT incubation the solution was transferred to cells (1 mL per well). Polyethylenimine (PEI) transfection was accomplished by mixing 3 µg pEGFP-C1 with 9 µL PEI (1 µg/µL, Polysciences Inc.) in 200 µL DMEM high without serum. After 15 min at RT, 800 µL of medium without serum were added and the transfection solution was transferred to cells. For Fugene, LTX, and PEI transfection, after 6 h the medium was removed from the wells and replaced with fresh EMEM with 10% FBS.

ASSCs from a subconfluent 25 cm^2^ flask were also electroporated (Equibio apparatus; 300 V, 25 µF, 240 V, 1050 µF, and 481 R; Opty-Puls) with 10 µg of pEGFP-C1 DNA in DMEM high with 10% FBS. Electroporated cells were transferred to new 25 cm^2^ flask and fed with complete medium; after 6 h the medium was replaced with fresh complete medium.

Transfected cells were incubated at 37°C with 5% CO_2_ in air in a humidified incubator and after 48 h cells were detached with trypsin, washed twice with sterile PBS and resuspended in 500 µL of PBS. Twenty five µL of each sample were analysed by the Tali image based cytometer (Invitrogen).

### Viruses and viral replication

Bovine herpesvirus-4 (BoHV-4, Movar strain), BoHV-4-A-EGFPΔTK [10], bovine herpesvirus-1 (BoHV-1, strain Oregon), bovine viral diarrhea virus (BVDV, strain NADL) and caprine herpesvirus-1 (CpHV-1, ATCC VR-462-1), were propagated by infecting confluent monolayers of MDBK or BEK cells at a multiplicity of infection (m.o.i.) of 0.5 50% tissue culture infectious doses (TCID_50_) per cell and maintained in MEM with 2% FBS for 2 h. The medium was then removed and replaced by fresh MEM containing 10% FBS. When approximately 90% of the cell monolayer exhibited cytopathic effect (CPE) (approximately 72 h post-infection), the virus was prepared by freezing and thawing cells three times and pelleting the virions through 30% sucrose, as described previously [11]. Virus pellets were resuspended in cold MEM without FBS. TCID_50_ were determined in BEK or MDBK cells by limiting dilution.

To test ASSCs susceptibility to these viruses, 3×10^5^ cells per well were seeded in 6 well plates; after 4 h cells were infected with a m.o.i. of 0.1 TCID_50_ of BVDV, BoHV-1, BoHV-4-A-EGFPΔTK and of 10^−3^ TCID_50_ of CpHV-1. Cells were monitored periodically for 96 h at the microscope to verify the CPE appearance and small aliquots of medium were recovered at 24, 48, 72 and 96 h and titrated in permissive cells, BEK for BoHV-4-A-EGFPΔTK, and MDBK for CpHV-1, BoHV-1 and BVDV. Forty-eight h after BoHV-4-A-EGFPΔTK infection, the cells were also detached and analysed by the Tali image based cytometer to verify the infection rate.

### Neospora caninum infection

Neospora caninum (Nc-Liverpool) strain was obtained from Professor S. Trees (University of Liverpool) and was propagated in VERO cells [12]. Parasites were harvested from their feeder cell culture and purified as described previously [13]. The number of tachyzoites was estimated using a haemocytometer. The final volume of suspension was adjusted with culture medium to achieve a ratio of 1∶1 parasite/host cell for subsequent infection experiments. Parasite viability was also checked using Alamar Blue assay and only preparations with greater than 95% viability were used.

### Lentivirus transduction and tumorigenicity assay

ASSCs at the 60^th^ passage were detached and 1×10^5^ cells were seeded in a 25 cm^2^ flask and left to attach for 4 h; then the culture medium was replaced by medium containing 2 transducing units (T.U.) per cell of a self-inactivating replication-incompetent third generation lentiviral vector expressing EGFP and luciferase (Lentiluc) under the control of the hEF1α promoter [14], [15] in 2 mL of complete medium. After 24 h the medium was replaced with 5 mL of fresh complete medium with 10% FBS and incubated at 37°C with 5% CO_2_ in air in a humidified incubator. Forty-eight h post transduction an aliquot of transduced cells was resuspended in PBS and subjected to analysis with Tali image based cytometer to assess transduction rate. The transduced cells were then maintained in culture and detached and resuspended in medium without FBS and inoculated into nude mice. Mice were imaged using bioluminescence (BLI) after 1, 7, 14, 21, 28 and 35 days following intraperitoneal injection of 150 mg/kg luciferin. The mice were anesthetised with gas anaesthesia (isoflurane 2.5%) and imaged for 5, 10, and 15 min after luciferin injection in order to minimize the pharmacokinetic variability among mice. Photons emitted from specific regions were quantified using Living ImageH software (Caliper Life Sciences).

### BoHV-4 infection and in vivo image analysis

ASSCs were seeded in a 6 well plate (3×10^5^ cells/well and infected with 1, 0.1 or 0.01 T.U./cell of Lentiluc. Forty-eight h after infection the cells were analysed through in vivo imaging, performed using an IVIS imaging system (Caliper Life Sciences). Cells were then detached, counted and inoculated intravenously, 5×10^5^ cells per mouse; mice were imaged using BLI after 4, 8, 12, 24 and 72 h following intraperitoneal injection of 150 mg/kg luciferin. The mice were anesthetised with gas anaesthesia (isoflurane 2.5%) and imaged for 5, 10 and 15 min after luciferin injection. Photons emitted from specific regions were quantified using Living ImageH software (Caliper Life Sciences) [16].

### Experimental Animals

Female inbred FVB (7–8 week-old) used for in vivo bio-distribution and nude mice used for the tumorigenicity assay were purchased from Harlan Laboratories Italy. Animals were maintained under conventional housing conditions. Prior to use, animals were acclimatized for at least 5 days to the local vivarium conditions (RT: 20–24°C; relative humidity: 40–70%), having free access to standard mouse chow and tap water. The experiments comply with the Principles of Animal Care (publication no. 85–23, revised 1985) of the National Institutes of Health and with the current law of the European Union and Italy (D. L.vo 116/92). The present project was approved by the Ethical Committee of the University of Parma (N∧38/13, 28/09/2013).

### Statistics

Data were analysed using one way analysis of variance (ANOVA) followed by Dunnet’s post hoc test for group comparisons. Results are reported as mean ± SD and significance attributed when P<0.05 (*) or P<0.01 (**).

## Results

### Generation of an alpaca skin cell line

**Figure 1:**
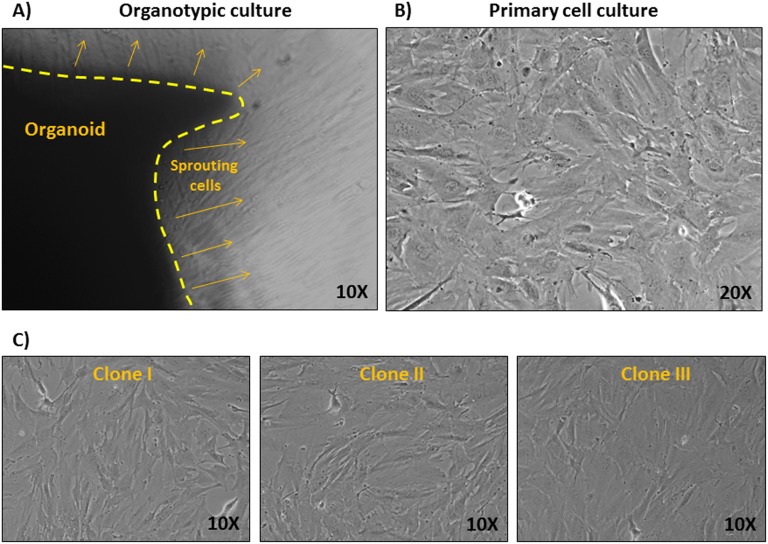
Generation of Alpaca organotypic culture. A) Representative phase contrast image (10x) of an Alpaca skin organoid, delimited by a yellow dotted line, with sprouting cells as indicated by yellow arrows. B) Representative phase contrast image (20x) of the first passage primary cell culture obtained from the cells sprouting from the organoids. C) Representative phase contrast images (10x) of three independent clones of cells after immortalization with SV40 large T antigen.

Before the generation of an alpaca immortalized cell line, a primary cell culture from an alpaca skin organotypic culture was established. An alpaca skin punch biopsy was cut in several pieces, placed in a plastic dish and incubated in a cell culture incubator in the presence of complete growth medium to allow the organoids to adhere to the plastic. Alpaca adherent skin organoids were kept in culture for one week, replacing the medium daily until cells emerged from the organoids (Fig. 1A). Organoids were removed and cells left to grow till they reached semi-confluency, then cells were trypsinized and passed to a flask. Thus, a first passage primary alpaca skin cell culture was obtained (Fig. 1B). Second passage alpaca skin cells were electroporated with a plasmid construct expressing SV40 large T antigen and stable transfected clones were selected for G418 resistance. Three clones were chosen on the basis of their morphology most closely reassembling the starting primary culture (Fig. 1C). However, a single clone (clone I) was used for further analysis.

### Alpaca cell line characterization

**Figure 2:**
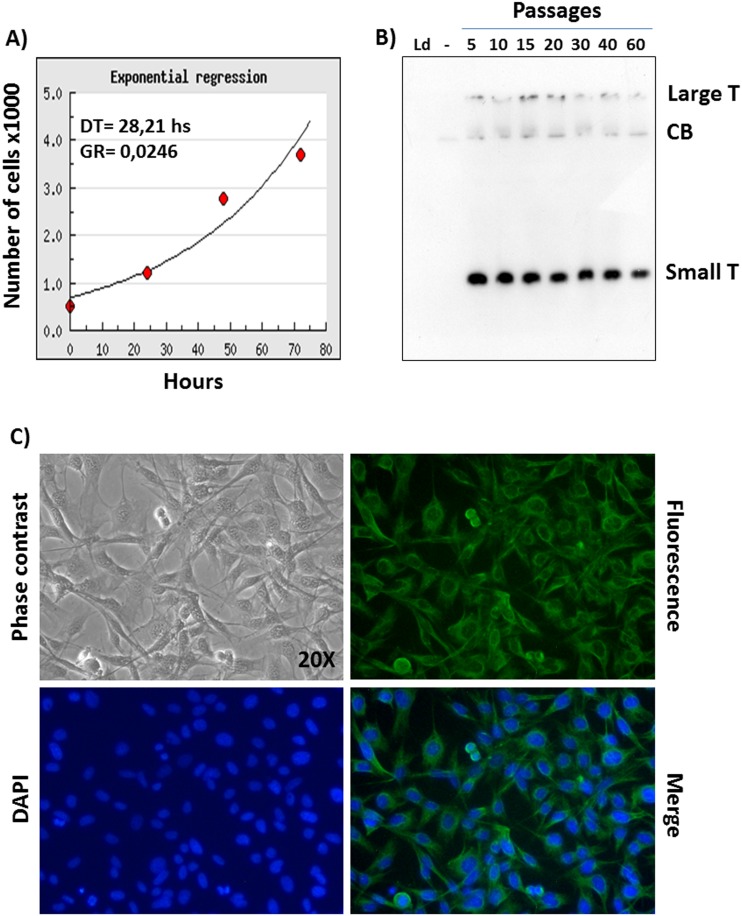
Cell line characterization. A) Exponential regression curve by which ASSCs doubling time (DT) and growth rate (GR) was calculated. B) SV40 large and small T antigen expression in ASSCs at different passages (5, 10, 15, 20, 30, 40 and 60) as monitored by Western immunoblotting. A cross reactive protein band (CB) is detected also in the negative control (−), ASSC primary culture. The ladder lane is indicated by Ld. C) Phase contrast and fluorescent images (20x) of ASSCs from the 60^th^ passage indicating expression of the stromal marker, vimentin. Counterstained nuclei with 4′, 6-diamidino-2-phenylindole (DAPI) were merged with green fluorescent image (merge). ASSCs from the 3^rd^, 10^th^, and 20^th^ passage stained similarly with α-vimentin antibody (not shown).

Sixtieth passage cells, corresponding to the passage number where a cell line can be considered immortalized, were assessed in terms of doubling time and growth rate. Using an equation for exponential regression, the doubling time was estimated to be 28.2 h and the growth rate 0.0246 doublings/h (Fig. 2A). Furthermore, SV40 large T immortalizing antigen was found to be consistently expressed through the passages, as monitored by western immunoblotting (Fig. 2B). Because this alpaca cell line was established from an alpaca skin biopsy, containing mainly stromal and epithelial cells, it was of interest to know the histological nature of these cells. Cells coming from several passages (3rd, 10th, 20th and 60th) were immuno-stained for keratin 14 and 18 (epithelial markers) or vimentin (stromal marker). A convincing, strong, positive immuno-staining signal was obtained only for vimentin (Fig. 2C), whereas no signal was obtained for keratins (data not shown). This result indicated a stromal origin of the cells and therefore this cell line was called Alpaca Skin Stromal Cells (ASSCs).

### ASSCs are permissive to ruminant DNA and RNA viruses

**Figure 3:**
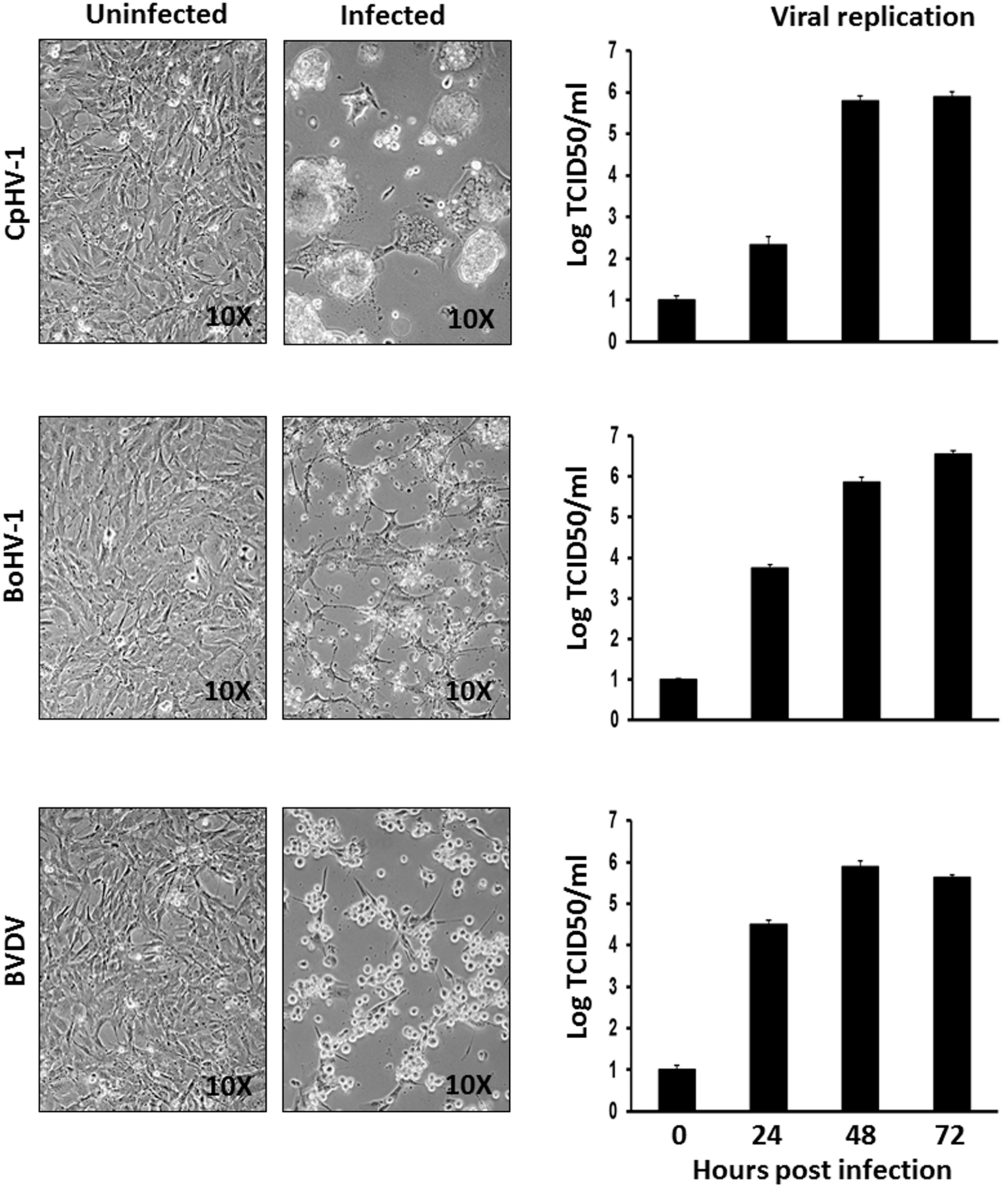
ASSC viral infection. Representative phase contrast images (10x) of uninfected and infected (CpHV-1, BoHV-1 and BVDV) ASSCs 48 h post infection, along with the replication kinetics of the respective viruses expressed as log TCID_50_/ml. The data presented are the means ± standard errors of triplicate measurements (*P*>0.05 for all time points as measured by Student’s *t* test).

Alpaca susceptibility to ruminant viruses has been documented [17]–[19]. The employment of a proper cell line, homologous to the pathogen host, is a very important issue when a virus has to be isolated, characterized and used for vaccine production. Therefore, the susceptibility of ASSCs to two DNA viruses (CpHV-1 and BoHV-1) and an RNA virus (BVDV) was tested. ASSCs were permissive to CpHV-1, BoHV-1 and BVDV as shown by the increase of the viral titre during the time post infection and the appearance of cytopathic effect (Fig. 3).

### ASSCs are permissive to Neospora caninum infection

**Figure 4:**
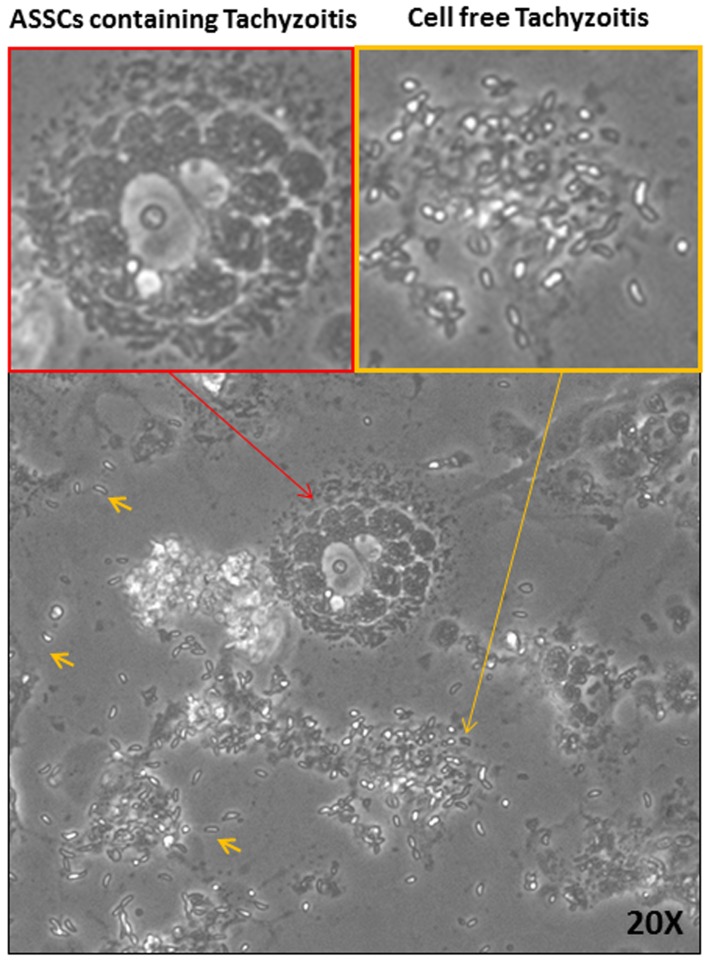
*Neospora caninum* infection of ASSCs. Representative phase contrast image (20x) of ASSCs containing *Neospora caninum* tachyzoites and cell free *Neospora caninum* tachyzoites at 48 h post infection.

*Neospora caninum is* a protozoan parasite found to be able to infect llamas and alpacas [20]–[22]. Therefore, as was investigated for viruses, it was of interest to know if ASSCs could support growth of *Neospora caninum* tachyzoites. ASSCs were infected with *Neospora caninum* tachyzoites and microscopically monitored daily. After 4 days post infection, large organized vacuoles containing *Neospora caninum* tachyzoites appeared in the cells and after 5–6 days the vacuoles containing tachyzoites began to become disorganized, releasing the tachyzoites into the cell culture medium (Fig. 4). Indeed ASSCs supported *Neospora caninum* tachyzoite growth.

### ASSCs can be efficiently transfected

**Figure 5:**
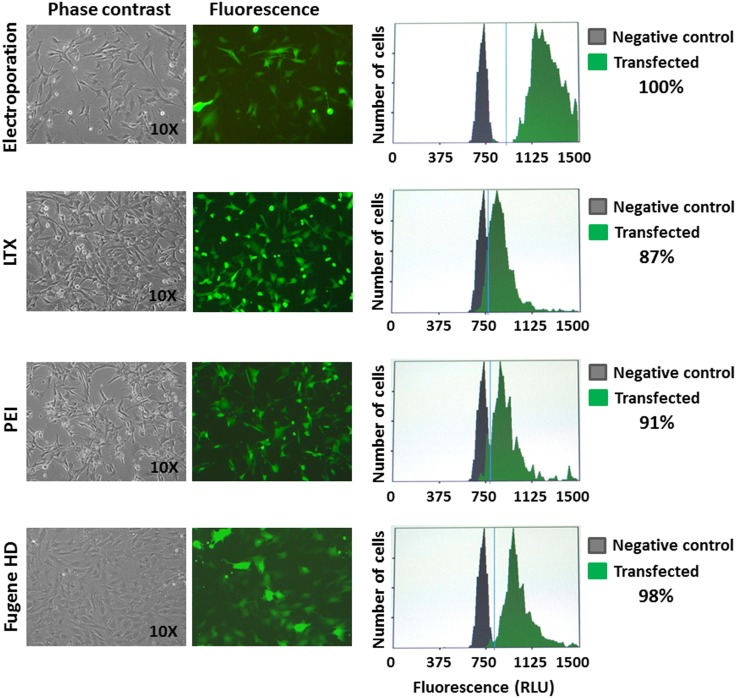
Transfection. Representative phase contrast and fluorescence images (10x) of ASSCs transfected with a GFP expression plasmid by different methods (Electroporation, LTX, PEI and Fugene HD), along with the efficiency of transfection as measured by cytometry. Each set of transfections was performed three times and standard deviations were negligible.

The ability to introduce foreign genes into a particular cell line is very important for both basic and applied studies. Therefore ASSCs were transfected by different methods [LTX (Invitrogen), PEI (Polyscience), Fugene HD (Promega) and electroporation] and the efficiency of transfection determined by image based-cytometry. Although ASSCs could be transfected very well by all methods used, the best efficiency was obtained by electroporation, which reached 100% transfection rate (Fig. 5).

### ASSCs can be transduced

**Figure 6:**
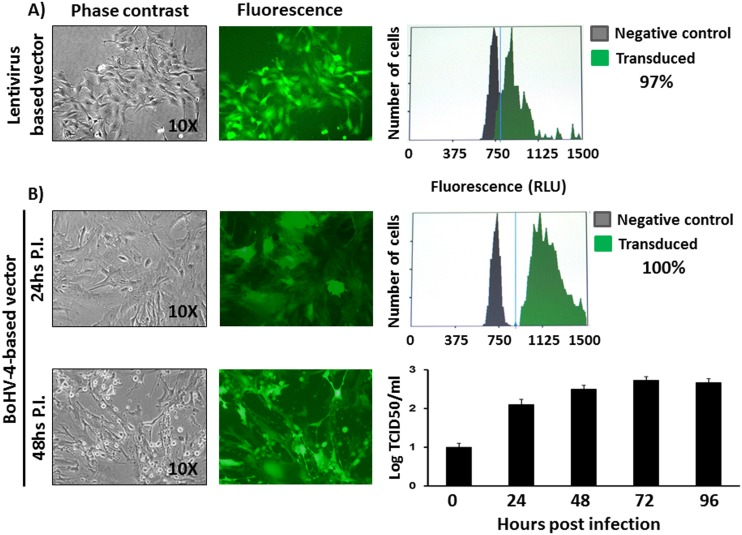
ASSC transduction. Representative phase contrast and fluorescence images (10x) of ASSCs transduced by a self-inactivating replicating incompetent third generation lentiviral vector at 48 h post transduction (A) and a replicating competent BoHV-4-based vector (B) at different times: 24 and 48 h post infection (P.I.). Efficiency of transduction was measured by cytometry. The titres of BoHV-4-based vector were examined by determining the numbers of cell free viruses through time (expressed as log TCID_50_/ml). The data presented are the means ± standard errors of triplicate measurements (*P*>0.05 for all time points as measured by Student’s *t* test).

As a first attempt to transduce ASSCs, two different viral vectors were employed: a lentiviral vector (Lentiluc) expressing both luciferase enzyme and green fluorescent protein through an internal ribosomal entry site (IRES), and a replication-competent bovine herpesvirus 4 (BoHV-4)-based vector expressing EGFP under the control of human cytomegalovirus immediate early promoter (hCMV) [10], [23]–[25]. ASSCs were infected with each vector and efficiency of transduction was monitored at 24 and 48 h post infection by image based-cytometry. Although both viral vectors transduced ASSCs with a very high efficiency, 100% for the BoHV-4-based vector (Fig. 6B) and 97% for the lentiviral vector (Fig. 6A), ASSCs were highly permissive for BoHV-4-based vector replication (Fig. 6B). In fact, a strong CPE developed in ASSCs as early as 48 h post BoHV-4-based vector infection, leading to a high cell free virus titre (expressed as logTCID_50_/ml). In contrast, lentiviral transduced ASSCs were grown for several passages, and the transgene was constantly expressed for all passages tested, without detrimental effects to the cells (results not shown).

### Labelled ASSCs allow bio-distribution tracking in mice

**Figure 7:**
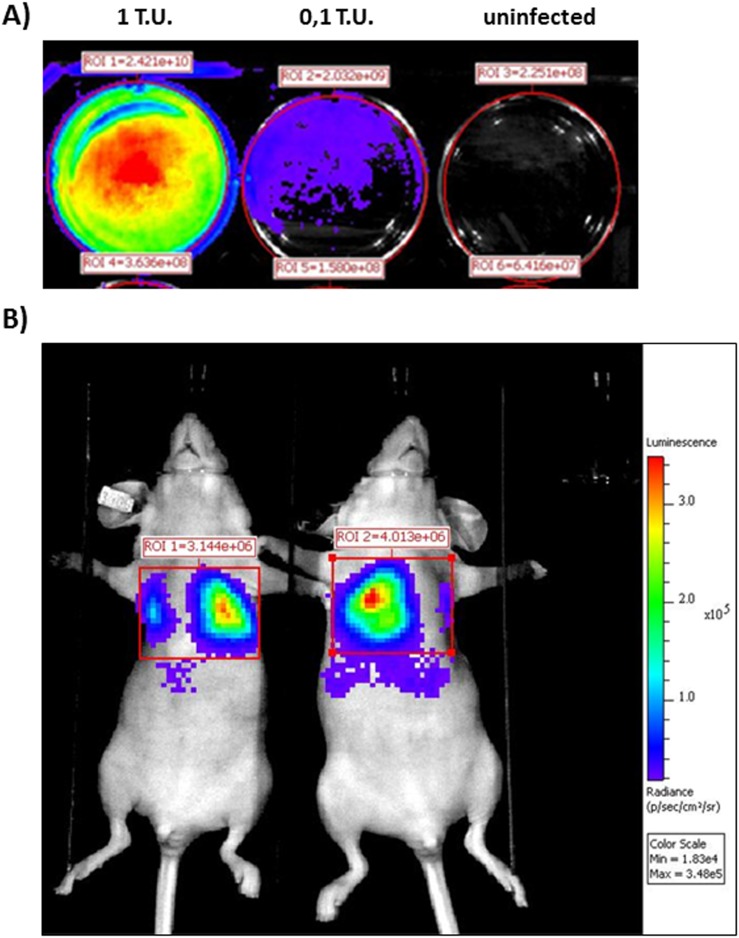
ASSC biodistribution. A) Representative in vivo bioluminescence image of ASSCs transduced with different doses (1 and 0.1 transducing units (T.U.)/cell) of a lentiviral vector expressing luciferase. B) Representative *in vivo* bioluminescence image of FVB mice displaying ASSCs transduced with a lentiviral vector expressing luciferase and localized to the lung 4 h post injection.

Traveling of compounds of interest after their injection in an experimental animal or human subject is called bio-distribution [26]. ASSC monolayers were infected with a lentiviral vector expressing luciferase, Lentiluc. To better investigate the correlation between Lentiluc infection and the bioluminescence signal, ASSCs cells were infected with different doses of Lentiluc (1 and 0.1 T. U./cell) and bioluminescence was measured 48 h post infection. Infected cells strongly expressed luciferase as monitored by in vivo bioluminescence imaging (BLI) analysis (Fig. 7A). When Lentiluc/ASSCs were intra-venously injected into FVB mice, as early as 3 min post injection a strong luminescence signal was localized in the lung (Fig. 7B), indicating the localization of the injected cells to the lung. The luminescence signal remained localized to the lung for the entire period of observation, 3 days, although the signal intensity decreased with time (data not shown).

### ASSCs are not tumorigenic

**Figure 8:**
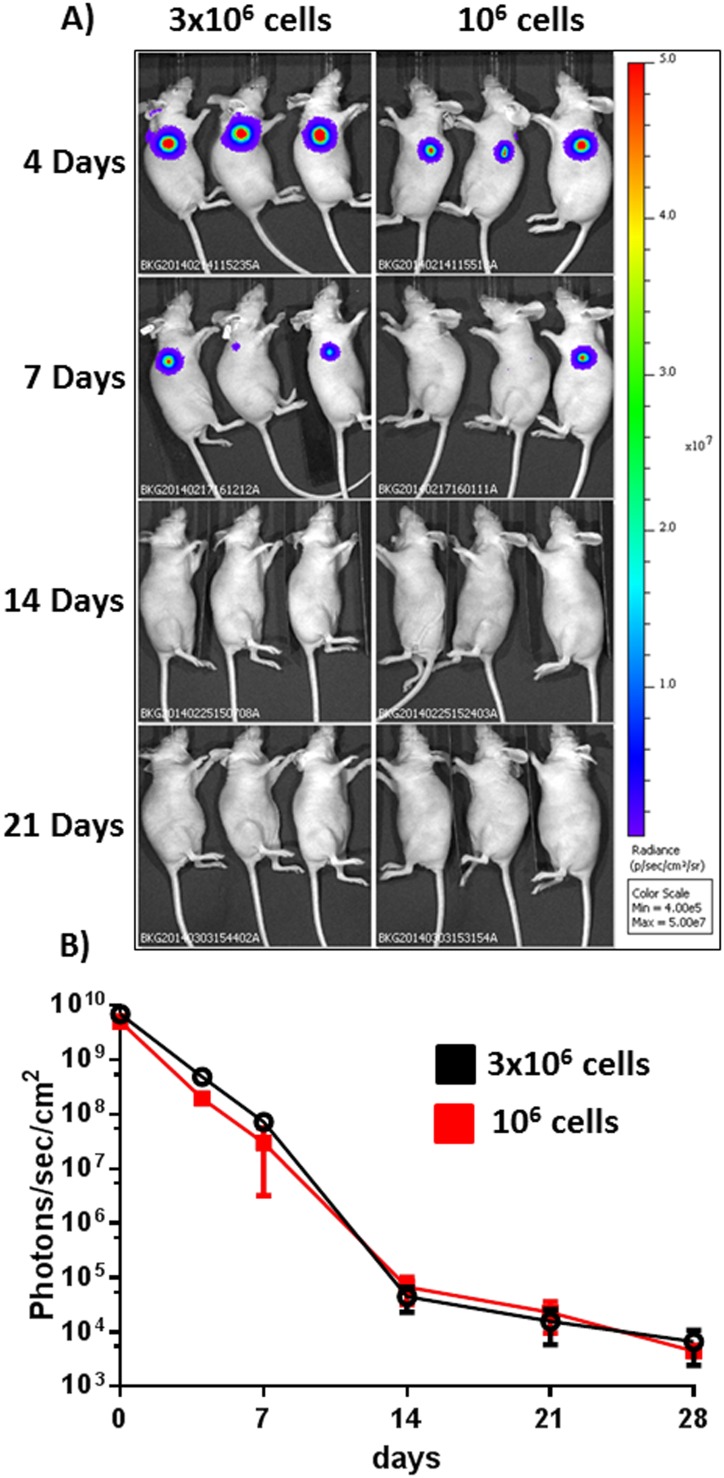
*In vivo* tumorigenesis assay. A) Representative in vivo bioluminescence images of nude mice subcutaneously inoculated with 3×10^6^ lentiviral transduced ASSCs on one side and 10^6^ on the opposite side of their body. Luminescence signal was acquired weekly until it was no longer detectable (B).

Because ASSCs were immortalized with an oncogenic antigen, SV40 Large T antigen, their potential tumorigenicity was a concern. The only way of testing whether a cell line has achieved all the characteristics needed for solid tumour growth is by testing its ability to form such tumours in vivo. In order to rule out the interference of the immune defence of the recipient, ASSCs were tested in nude mice [27]. A group of 6 nude mice were subcutaneously inoculated with two doses (10^6^ on one flank and 3×10^6^ on the other flank) of lentiviral stably transduced ASSCs expressing luciferase and monitored weekly by BLI for tumour formation. In all inoculated mice and for both doses of inoculated cells, tumour formation did not take place as observable by the complete extinction of detectable luminescence signal and complete absence of palpable solid tumours (Fig. 8A and B) by 28 days post inoculation. However the mice were kept under observation for other 10 weeks and the result did not change. Therefore, it could be stated that ASSCs are not tumorigenic, at least by the test used in this study.

## Discussion

Within and outside their country of origin, alpacas and domestic ruminants, such as cows, goats and sheep, have similar grazing patterns and diet. Domestic ruminants and alpaca have multiple opportunities for interspecies pathogen transmission, as observed for *Brucella abortus* [28], [29], bovine viral diarrhea virus [17]–[19], *Mycobacterium bovis* [3]–[5], etc. in North America. In multi-host systems, wildlife or domestic species may act as reservoir or spill-over hosts, or be part of the maintenance community, for a number of livestock pathogens. Among factors influencing the potential for interspecies pathogen transmission (related to pathogen biology, wildlife behaviour and ecology, and livestock management), the transmission route of the pathogen is of particular interest, and has previously been discussed as a factor related to the emergence of zoonotic pathogens. However, there is still much to learn about the relevance of transmission pathways in multi-host systems in general. Because pathogens with different transmission pathways may require very different prevention and control strategies, understanding the relative importance of transmission routes in multi-host systems is essential.

Tissue culture is used widely in laboratories involved in pathogen isolation and serial propagation, and primary cells, due to their viral susceptibility spectrum similar to that of the natural organism, are the laboratory host of choice despite the fact that they display several disadvantages. In order to reduce the adverse features they share, established cell lines, with well-defined and standardized properties, seem to be a promising and useful alternative biological system. In the present paper, the first stable immortalized cell line of Alpaca Skin Stromal cells, ASSCs, was established by transfection of primary alpaca skin stromal cells with a plasmid containing expression cassettes for the SV40 large T antigen and *neo* genes. One of the reasons that prompted us to generate an alpaca immortalized cell line was the necessity to have an in vitro substrate to isolate and replicate alpaca pathogens or micro-organisms potentially pathogenic to other animal species.

Virus isolation from clinical specimens is one of the main goals in diagnostic laboratories and it is generally carried out in cell cultures, in chicken embryos and, to a lesser extent, in laboratory animals [30]. Primary cultures, derived from animal tissues that have not undergone sub-culturing, have a wide susceptibility, mainly to viruses that infect the same species from which the cells were derived. However they have several disadvantages including the risk of latent virus contamination, the high cost involved in maintaining a continuous supply, the necessity to use cells from different tissues, less efficient recovery rate than that of continuous cell lines and no real possibility of standardization. Consequently, diploid cell lines, which can be sub-cultured for numerous passages, may provide safe, viable systems for either isolation of viruses from clinical specimens or for the production of biologicals. However, the use of such cell lines is controversial as they may have properties in common with cancer cells such as a modified karyotype or contamination with viral or transforming agents that could induce tumours in immuno-suppressed laboratory animals. However the advantages of established cell lines include an infinite life span allowing their characterization at pre-determined passages, the possibility to clone them easily, ability to be adapted to grow in particular culture media and the possibility to easily document their life history. Accordingly, continuous cell lines are attractive candidates for pathogen isolation and serial in vitro propagation. Indeed ASSCs generated in this work were able to propagate domestic ruminant viruses and tachyzoites of the intracellular parasite *Neospora caninum*. Furthermore, they were not tumorigenic when inoculated into immunodeficient mice.

In members of the family *Camelidae*, which includes alpacas, a large proportion of the humoral immune response is comprised of homodimeric immunoglobulin G (IgG) (∼80 kD) devoid of light chains [31]. These heavy-chain antibodies also lack the C_H_1 region, and their variable region is referred to as VHH or nanobodies. Recombinant nanobodies (∼14 kD) are intact antigen-binding domains and exhibit a broad antigen-binding repertoire. They have unique characteristics, including an extended complementarity-determining region 3 (CDR3) loop that can adopt a protruding conformation allowing interaction with concave epitopes that are occluded for conventional antibodies [32]. To stabilize the enlarged CDRs, nanobodies often possess an additional disulfide bond between CDR1 and CDR3 in dromedaries, and CDR2 and CDR3 in llamas and alpaca [33]. Nanobodies to numerous antigens have been produced [34] and despite being monovalent, they frequently exhibit biological activities comparable to conventional bivalent antibody molecules [35]. As such, nanobodies are a promising tool for targeted immunotherapy. Therefore, an efficient system for alpaca immunization is an important issue for nanobody generation. Thus, ASSCs were proposed as suitable antigen carriers for alpaca immunization. The most desirable features of cells to be used as antigen carriers for immunization purposes are: ease of preparation and expansion as cell culture, susceptibility to transfection and transduction, and high-level foreign antigen expression. Most of the above requirements are potentially met by ASSCs. In fact ASSCs were easily transfectable by physical and chemical methods as well as transducible by viral vectors. Further, after intravenous infusion into mice of ASSCs stably transduced with Lentiluc, bioluminescence signals associated with the stably transduced ASSCs were detected mainly in the lungs. This is probably because of the small size of the pulmonary capillaries and the larger size of the cells. Similar results were obtained with other cell types such as mesenchymal stem cells [36].

The most remarkable characteristic of ASSCs was their strong susceptibility not only to a self-inactivating replication incompetent lentiviral vector but also to a replication competent BoHV-4-based vector. Bovine herpesvirus 4 (BoHV-4) is a dsDNA virus belonging to the *Herpesviridae* family, *γ-herpesviridae* subfamily [37]. Although replication of most γ-herpesviruses is restricted to their natural host species, BoHV-4 is highly promiscuous. It has been recovered from a variety of ruminants besides cattle, plus sporadically isolated from species as diverse as lions, cats and owl monkeys [38], [39]. BoHV-4 has been reported to infect goats [40], [41], guinea pigs, and rabbits [42]. The construction of infectious, BoHV-4-derived Bacterial Artificial Chromosome (BAC) clones in *Escherichia coli*, followed by progeny virion reconstitution via transfection into permissive eukaryotic cells, has been successfully pursued as a means to rapidly modify the viral genome and adapt it to specific needs [43], [44]. Animals such as rabbits, sheep, goats and chickens have been successfully immunized with recombinant BoHV-4-BAC derivatives expressing heterologous antigens [41], [43]–[45]. We thus want to emphasize the potential use of BoHV-4-vectorialized foreign antigens as prototype recombinant vaccines either for direct, BoHV-4-based vector injection, or indirect, by BoHV-4-based vector preloaded into ASSCs, alpaca immunization. This proof of concept was accomplished in a previous study, where swine adipose-derived stromal cells (SADSC) were chosen as carriers for BoHV-4 virions delivering heterologous antigens [46]. A stronger humoral immune response was obtained for SADSCs delivering recombinant BoHV-4 compared to recombinant BoHV-4 alone. The promising results and the proof-of-concept on the use of ASSCs as antigen carriers for alpaca immunization, along with a BoHV-4-based vector, will pave the way for the production and testing of additional prototype immunogens for the generation of nanobodies in alpaca. Although ASSCs are fully permissive to BoHV-4-based vector replication, and CPE could be a limitation in vitro, in vivo it could be instead considered a benefit. Parenteral inoculation of ASSCs infected with recombinant BoHV-4s expressing antigen would lead to: the expression of the antigen in loco, ASSC lysis due to BoHV-4 replication, cross infection of surrounding cells and high-level expression of the foreign antigen(s), followed by an enhanced immune-response and ultimately a better immunization.
